# Feasibility, acceptability and impact of integrating malaria rapid diagnostic tests and pre-referral rectal artesunate into the integrated community case management programme. A pilot study in Mchinji district, Malawi

**DOI:** 10.1186/s12936-016-1237-2

**Published:** 2016-03-21

**Authors:** Themba B. Phiri, Blessings N. Kaunda-Khangamwa, Andrew Bauleni, Tiyese Chimuna, David Melody, Humphreys Kalengamaliro, John H. Sande, Humphreys Kampira Nsona, Don P. Mathanga

**Affiliations:** Malaria Alert Centre, College of Medicine, Blantyre, Malawi; Save the Children Malawi, Lilongwe, Malawi; Ministry of Health, Lilongwe, Malawi

**Keywords:** Pre-referral rectal artesunate, Hard to reach areas, Malaria rapid diagnostic tests, Feasibility, Correct performance of RDTs

## Abstract

**Background:**

The World Health Organization recommends that persons of all ages suspected of malaria should receive a parasitological confirmation of malaria by use of malaria rapid diagnostic test (RDT) at community level, and that rectal artesunate should be used as a pre-referral treatment for severe malaria to rapidly reduce parasitaemia. This paper reports on findings from a pilot study that assessed the feasibility, acceptability and effects of integrating RDTs and pre-referral rectal artesunate into the integrated Community Case Management programme in Malawi.

**Methods:**

This study used mixed methods to collect information for this survey. Pre- and post-intervention, cross-sectional, household surveys were carried out. A review of integrated community case management reports, including supervision checklists was conducted. Quantitative data were collected in tablets running on open data kit software, and then data were transferred to STATA version 12 for analysis. For key indicators, proportions were calculated at 95 % confidence intervals. Qualitative data were recorded onto digital recorders, translated into English and transcribed for analysis.

**Results:**

Out of 86 observed RDT performances, a total of 83 (97 %) were performed correctly with a proper disposal of sharps and biohazard wastes. Only two (2 %) febrile children who had an RDT negative result were treated with artemether–lumefantrine, contrary to malaria treatment guidelines. Utilization of community health workers (CHWs) as a first source of care increased from (33.9 %) (95 % CI; 25.5–42.3) at baseline to (89.7 %) (95 % CI; 83.5–95.5) at end line in the intervention villages. There was a corresponding decrease in the proportion of caregivers that first sought care from informal sources from 12.9 % (95 % CI; 6.9–18.9) to 1.9 % (95 % CI; 0.9–4.4) in the intervention villages. Acceptability of the use of RDTs and pre-referral rectal artesunate at the community level was relatively high.

**Conclusion:**

Integration of RDTs and pre-referral rectal at artesunate community level is both feasible and acceptable. The strategy has the potential to increase and improve utilization of child health services at community level. However, this depends on the CHWs’ skills and their availability in remote areas.

## Background

The World Health Organization (WHO) now recommends that persons of all ages suspected of malaria should receive a parasitological confirmation of malaria by either a malaria rapid diagnostic test (RDT) or microscopy [1]. As a result, RDTs are now being integrated into malaria community case management programmes [2–4] with an assumption that integration will reduce waste and mitigate against resistance [5]. Studies have shown that rectal artesunate can be used as a pre-referral treatment of severe malaria to rapidly reduce parasitaemia and mortality [6]. The availability of this pre-referral treatment at community level would reduce deaths as children move through the referral system from community health workers (CHWs) to health centre where they can access parenteral treatment.

In Malawi, infant mortality (66/1000) and under five mortality rates (112/1000) remain unacceptably high. The majority of the deaths are due to malaria, pneumonia and diarrhoea. As in most of sub-Saharan Africa (SSA), in Malawi children under the age of 5 years bear the highest burden of malaria, accounting for nearly 50 % of the new cases recorded between 2005 and 2009. In 2010, 35 % of children under age of five had a fever, presumably malaria, in the 2 weeks preceding the survey [7]. Among these children, only 28 % were given effective anti-malarial drugs promptly (same day or the day following the onset of the fever) whilst 35 % sought treatment outside the formal health system. Since 2008, Malawi has implemented an integrated Community Case Management (iCCM) programme, which is managed by trained Health Surveillance Assistants (HSAs). HSAs are government employed community based health workers that receive an average salary of US $60.00 per month. This cadre of health workers has existed within the Ministry of Health structure for more than 20 years. HSAs are purposefully tasked to reside within hard-to-reach villages and serve children <5 years of age [8]. In total, there are 3622 hard-to-reach areas in Malawi. Hard-to-reach areas in Malawi are defined as areas that render difficulties in communication and access with the nearest health facilities. There are a variety of factors that come into play for an area to be described as hard-to-reach such as distance from nearest health facility of not less than 8 km, existence of a barrier such as a mountain between the village and the health facility, poor road that is inaccessible or difficult to access during the rainy seasons between the health facility and the village (area) just to mention a few. As of September 2015, over 4500 CHWs from all hard-to-reach areas and those that are allocated at district hospitals and health centres had received iCCM training, and were providing services nationwide at community level either from their houses or any community agreed structure.

In 2013, the new Malawi national malaria treatment policy recommended that RDTs and pre-referral rectal artesunate be integrated into the community case management of malaria. This paper reports on findings from a pilot study whose aim was to assess the feasibility, acceptability and effects of integrating RDTs and rectal artesunate into the Malawi iCCM. The study findings generated evidence to guide scale-up of the integration of RDTs and pre-referral rectal artesunate in Malawi and other malaria-endemic countries.

## Methods

### Study site

This pilot study was conducted in Mchinji district, a district in the central region of Malawi with a population of 511,792. Mchinji is a rural district with high malaria prevalence, relatively low insecticide-treated net (ITN) coverage and high under-five (119 per 1000 live births) and infant (57 per 1000 live births) mortality rates. CHWs serving hard-to-reach areas of the district provide iCCM services to children aged 2–59 months. In 2012, 87 % of all hard-to-reach communities in the district [9] had a CHW trained to treat common childhood diseases (malaria, pneumonia and diarrhoea). In 2014, the district implemented a pilot project in selected hard-to-reach villages to integrate RDTs and pre-referral rectal artesunate into the iCCM programme. Selected CHWs from the intervention hard-to-reach areas only received an additional three-day refresher training which included modules on how to perform RDTs, appropriately treat febrile children based on RDT results, assess for danger signs and administer rectal artesunate. CHWs from the control area did not receive this additional 3 days training and were not supplied with RDTs and RA. The three main objectives of this refresher training were; to improve HSAs skills in the performance of RDTs, to impart skills of administering rectal artesunate among HSAs and to improve HSAs skills in disposal of bio wastes generated from use of RDTs and RA. This refresher training also covered other aspects of iCCM, including management of fast breathing, diarrhoea, routine assessment of anaemia, and malnutrition in sick children. To encourage the use of the services, community meetings were undertaken in the selected villages to inform caretakers of the introduction of RDT and pre-referral rectal artesunate, explain the procedures and answer any questions or concerns. In addition to the normal iCCM drugs (artemether–lumefantrine, amoxicillin, zinc and oral rehydration solution), trained and certified CHWs received RDTs and rectal artesunate from the nearest health facility.

### Study design and data collection

The feasibility of implementing the programme and the quality of care provided (technical performance in assessing, classifying, treating sick children with the support of RDTs, and the use of rectal artesunate) was assessed. Forty CHWs who administered RDTs and pre-referral rectal artesunate were included in the sample and were paid unannounced visits for data collection purposes. The quality of care assessment was based on standard integrated management of childhood illnesses (IMCI) evaluation methods and involved the review of treatment registers, direct observation of all CHWs assessing sick children, re-examination of the child and conducting exit interviews with the caretakers. The data collection forms were based on the IMCI health facility survey evaluation methods and included questions which were recently adapted and used to assess quality of care provided by CHWs in Malawi [10]. The indicators covered assessment, classification, treatment, counselling, safe disposal of biohazard waste materials and referrals. The performance of CHWs in using RDTs and administration of pre-referral rectal artesunate was also assessed using standard checklists.

To assess the acceptability of the programme, focus group discussions (FGD) and key informant interviews (KII) were conducted with CHWs, caretakers, including fathers of children, community leaders, and other key stakeholders involved in the programme, to understand the challenges and strengths of the programme. FGD’s and KII were employed to have a deeper understanding, experiences of both health workers and caregivers as well as to strengthen our key findings. Qualitative data were recorded onto digital recorders, translated into English and transcribed for analysis.

Pre- and post-intervention household surveys were carried out to provide information on the effect of providing malaria diagnosis and pre-referral treatment in the utilization of services at community level. Both surveys were conducted between the months of September and October 2013 and 2014, respectively. This provided an implementation period of 1 year between the two surveys. A three-stage random cluster sampling technique was used to sample households with children 2–59 months. The first stage involved dividing the district into two halves and randomly selecting one half as the intervention area and the other half as a control area. For the study. The second stage involved selection of hard to reach areas using probability proportional to size (PPS) approach. This sampling procedure involved listing all the hard to reach areas in the selected half of the district and then applying a cluster sampling methodology to select the twelve hard to reach areas.

In each selected area, all the households were listed through a census and 43 households with 556 and 705 children aged 2–59 months were enrolled to participate in both surveys, respectively. A household in this survey is defined as a person or a group of persons, related or unrelated who live together and share common cooking and eating arrangements. One caregiver was interviewed for each household selected to participate in the survey.

This study was approved by the College of Medicine Research and Ethical Committee (COMREC) with certificate of approval number P.02/13/1349. Informed written consent was obtained before conducting the interviews.

### Data analysis

Data for quantitative surveys (community cross-sectional surveys and assessment of quality of care) was collected electronically using tablets running on open data kit (ODK) software. For key indicators, proportions with 95 % confidence intervals were calculated. Where applicable, p values were calculated and reported. All analyses were performed in Stata 12.

The key informant interview and group discussion transcripts were arranged as condensed data and a coding framework was generated to capture dominant and distinct themes. Initial coding was done manually to identify specific pieces of data corresponding to different themes and entered into Microsoft Excel by an experienced social scientist. The social scientist identified recurrent themes, issues through content and thematic analysis to gauge dominant and distinct themes. Further, data analysis employed a triangulation approach in search of causality, related themes between the methods for data validation. The analysis was followed by extraction of quotes that represented consensus.

## Results

Table 1 presents data from iCCM reports (HMIS) from selected intervention hard-to-reach areas in Mchinji district between January and September 2014 and shows that RDTs were performed in a total of 19,732 new fever cases, of which 13,812 (70 %) were RDT positive. A total of 268 (1.4 %) children that were identified with one or more danger signs, were administered with pre-referral rectal artesunate and referred to the nearest health facility. iCCM supervision reports (checklists) between January and September 2014 show that 29 out of 31 (94 %) CHWs observed by district iCCM supervisors administering pre-referral rectal artesunate correctly conducted the procedure.

**Table 1:** Mchinji district CCM data (HMIS reports) showing use of RDTs and rectal artesunate in the 40 intervention hard-to-reach areas between January and September 2014

Month 2014	New fever cases seen at village clinics (with RDTs and RA)	Malaria confirmed cases (RDT positive)	Children with severe illness that were referred and that received RA
January	1758	1238 (70.4 %)	29
February	2308	1621 (70.2 %)	28
March	2422	1542 (63.6 %)	22
April	2253	1678 (74.4 %)	35
May	2680	1977 (73.7 %)	36
June	2231	1653 (74.1 %)	30
July	2217	1574 (71 %)	27
August	1844	1272 (69.1 %)	32
September	2019	1440 (71.3 %)	29
Total	19,732	13,995 (70.9 %)	268

### Quality of care survey conducted in the intervention hard-to-reach areas

#### Characteristics of Health Surveillance Assistants (HSAs) included in the quality of care survey

A total of 40 Health Surveillance Assistants were observed assessing and classifying sick children at the selected village clinics. Table 2 shows the characteristics of HSAs that participated in the study. The majority of HSAs were male 36 (90 %) with the mean age of 37.4 years and attended Malawi School Certificate of Education level (47.5 %). In terms of training and experience, all HSAs received HSA training and had an average of 10 years of experience working in the communities. The majority of HSAs 34 (85 %) received initial Community Case Management (CCM) training between 2010 and 2012. All HSAs surveyed had received training in malaria testing using RDTs, administration of pre-referral rectal artesunate and disposal of sharps and biohazard wastes 6 months before the survey.

**Table 2:** Characteristics of study HSAs (Community Health Workers) that participated in the quality of care survey in the intervention hard-to-reach areas

Variable	n (%)
CHWs (N = 40)	
Mean age of CHWs in years	37.4
Average years of experience working as CHWs in community	10.4
Sex (male)	36 (90.0)
Year CHWs received initial iCCM training	
2010–2012	34 (85.0)
2013–2014	6 (15.0)
CHWs trained in performance of RDTs and RA administration	40 (100.0)
Level of education	
Form 2	8 (20.0)
Form 4	19 (47.5)
Form 4 plus post basic training	12 (30.0)
Professional training	1 (2.5)

#### Performance of RDTs by HSAs in the intervention hard-to-reach areas

During the survey, 97.5 and 85 % of the sampled CHWs had RDTs test kits and rectal artesunate suppositories, respectively, and all of these were correctly stored in drug boxes. Table 3 shows that a total of 90 (75 %) out of 120 sick children observed during the survey had fever. CHWs were observed performing a total of 86 (96 %) RDTs among children with fever; of these, 83 (97 %) RDTs were performed correctly, including the proper disposal of sharps and biohazard waste. Only two (2 %) febrile children with RDT-negative result were treated with artemether lumefantrine, contrary to malaria treatment guidelines. During the quality of care survey, only three children had danger signs requiring rectal artesunate administration. Two out of three observations were correctly administered with pre-referral rectal artesunate.

**Table 3:** Proportions of children for whom specific case management tasks were performed by CHWs; quality of care survey

Variable	n/N (%)	95 % CI
Classification		
Children whose classification of uncomplicated fever given by CHWs matched the classification by IMCI trained clinician (Gold standard)	78/83 (93.9)	[85.8–99.7]
Children whose classification of uncomplicated diarrheoa given by CHWs matched the classification by IMCI trained clinician (Gold standard)	17/19 (89.4)	[79.3–97.0]
Children whose classification of uncomplicated cough/fast breathing given by CHWs matched the classification by IMCI trained clinician (Gold standard)	10/14 (71.4)	[62.1–82.4]
Treatment of child illness by CHWs		
Children with fever plus RDT +ve results who are prescribed an antimalarial (ACT) correctly	49/55 (89.1)	[75.3–96.9]
Children with uncomplicated cough/fast breathing who are prescribed an antibiotic correctly	9/14 (64.3)	[58.7–72.1]
Children with uncomplicated diarrheoa who are prescribed ORS correctly	13/16 (81.2)	[72.6–93.2]
Children with uncomplicated diarrheoa who are prescribed zinc correctly	5/6 (83.3)	[74.1–95.3]
Performance of RDTs by CHWs		
CHWs with RDTs available at their village clinic	39/40 (97.5)	[95.3–102.1]
Children with fever RDT performed	86/90 (95.5)	[83.2–99.8]
RDTs performed correctly by CHWs^a^	83/86 (96.5)	[86.8–101.8]
Children with RDT +ve results	48/83 (57.8)	[43.5–67.1]
RDT results adhered to by CHWs when providing malaria treatment	82/86 (95.3)	[74.8–93.7]
RDTs performances on which CHWs adhered to SOPs for disposal of bio-wastes and sharps	82/86 (95.4)	[85.7–101.2]
Referral for danger signs and use of pre-referral rectal artesunate		
Children with general danger signs referred	7/9 (78.0)	[58.7–89.2]

Three children presenting danger signs were observed being administered with rectal artesunate by HSAs during the assessment of care survey. Two out of these 3 (66.7 %) were observed to have been correctly administered with rectal artesunate suppository by HSAs

Correct administration of RA means CHWs being able to adhere to Standard Operation Procedures (SOPs) for administration of RA

^a^ Correct performance of RDTs means that CHWs followed the Standard Operation Procedures (SOPs) for performing RDTs including disposal of bio-wastes on the day of survey

### Qualitative survey

#### Community acceptance of RDTs and rectal artesunate

Ability in the use of RDTs and rectal artesunate at the community level was quite high. Most caregivers cited *“timely intervention”, “proper diagnosis,” “proper treatment”, “proper diagnostic tests”, “reducing travel distance”* as part of the benefits. Although the use of rectal artesunate was new in the community, caregivers were comfortable with the anal route of administration, likening it to receiving treatment for constipation. Despite the high acceptance among caregivers and community members, a key challenge for implementation of the programme is the location of CHWs, some of whom do not reside in the catchment area they are expected to serve, had consequently inflexible village clinic opening hours and only provided treatment on scheduled days.

### What has been the community experience with RDTs and rectal artesunate so far?

R3: The CHW refuses to treat the children on the days the village clinic is closed because he says he is busy or because he does not live in the village.
R4: Sometimes he treats children at night, but he mostly refers them. Some people are helped when they take their children to the CHW at night; some are not (FGD, KAI, 004, 2014).
R3: Our CHW doesn’t stay within the community, he only comes once or twice a week…
R5: The CHW is available during weighing period of under-five and he comes with malaria treatment if available during that time(FGD, KAP, cont, 003, 2014).

Other concerns raised by caregivers included: the effect of drawing blood in the community, apprehension that the tests might be for HIV, and the strict adherence to malaria policy by CHWs with a negative RDT meaning children were not given anti-malarials. Community members also complained that in most circumstances referrals were done without any referral slips, which meant that caregivers had to queue for treatment at the health facility, further delaying treatment of the severely sick child.

The CHWs and their supervisors indicated that the integration of RDTs and pre-referral rectal artesunate into iCCM improved access, testing and treatment, and also improved the referral system. CHWs felt that the provision of services that would normally be provided at health facility level boosted their confidence and elevated their standing in the community as ‘village doctors’, and in some cases led to the construction of shelters to be used as a village clinic. While CHWs confirmed accepting the RDTs and rectal artesunate (RA) services, they however mentioned that CCM services were scheduled only on certain days of the week due to other equally important duties they were expected to perform.

### Household surveys (baseline and post intervention assessments)

A total of 705 and 648 children aged 2–59 months were enrolled at baseline and post-intervention surveys, respectively. Utilization of CHWs as a first source of care increased from (33.9 %) (95 % CI; 25.5–42.3) at baseline to (89.7 %) (95 % CI; 83.5–95.5) at end-line in the intervention villages. In the control villages, the proportion increased slightly from (40.9 %) (95 % CI; 30.5–51.3) to (47.6 %) (95 % CI; 36.8–58.3). There was a corresponding sharp decrease in the proportion of caregivers who first sought care from health facilities and informal providers from 50.8 % (95 % CI; 41.9–59.7) to 7.5 % (95 % CI; 2.5–12.5) and from 12.9 % (95 % CI; 6.9–18.9) to 1.9 % (95 % CI; 0.9–4.4), respectively, in the intervention villages. While in the control villages, slight decrease was observed in the proportion of caregivers who sought care from health facilities and informal providers from 36.45 (95 % CI; 26.2–46.5) to 32.1 % (95 % CI; 22.0–42.2) and from 21.6 % (95 % CI; 12.9–30.3) to 15.5 % (95 % CI; 7.6–23.2) respectively. Overall, survey results show more positive changes in the sites where RDTs and pre-referral rectal artesunate were integrated into the iCCM programme when compared with both the control villages and baseline values (Table 4).

**Table 4:** Utilization of village clinics for child’s illness, use of medicines at village clinics managed by CHWs in Mchinji district 2014: data collected at baseline survey (October 2013) and end line survey (October 2014)

Variable	Intervention		Control	
	Baseline	End line	Baseline	End line
	n (%) [95 % CI]	n (%) [95 % CI]	n (%) [95 % CI]	n (%) [95 % CI]
Source where care for child’s illness was sought first	(N = 124)	(N = 107)	(N = 88)	(N = 84)
CHWs (village clinics)	42 (33.9) [25.5 to 42.3]	96 (89.7) [83.9 to 95.5]	36 (40.9) [30.5 to 51.3]	40 (47.6) [36.8 to 58.3]
Government Health Facility	63 (50.8) [41.9 to 59.7]	8 (7.5) [2.5 to 12.5]	32 (36.4) [26.2 to 46.5]	27 (32.1) [22.0 to 42.2]
Mission (CHAM) Health Facility	3 (2.4) [0.03 to 5.0]	1 (0.9) [0.7 to 2.7]	1 (1.1) [−1 to 3.4]	4 (4.8) [0.1 to 9.3]
Other non-formal sources (shops, traditional healers)	16 (12.9) [6.9 to 18.9]	2 (1.9) [0.9 to 4.4]	19 (21.6) [12.9 to 30.3]	13 (15.5) [7.6 to 23.3]
Use of LA at village clinics	(N = 30)	(N = 36)	(N = 19)	(N = 15)
Children with fever/confirmed malaria that received LA	18 (60.0) [41.4 to 78.6]	35 (97.2) [91.8 to 104.3]	14 (73.6) [47.9 to 92.7]	14 (93.3) [76.3 to 98.4]
Use of ORS and zinc at village clinics	(N = 20)	(N = 19)	(N = 12)	(N = 15)
Children with diarrhoea that received ORS	17 (77.3) [58.3 to 96.3]	12 (63.2) [41.8 to 89.9]	7 (58.5) [44.3 to 69.4]	10 (66.6) [37.4 to 87.5]
Children with diarrhoea that received Zinc	2 (8.3) [3.5 to 20.3]	10 (52.6) [46.2 to 78.1]	4(20.0) [0.8 to 39.2]	9 (60.0) [46.5 to 76.2]
Use of antibiotics at village clinics	(N = 24)	(N = 41)	(N = 6)	(N = 10)
Children with cough that received Antibiotics	21 (84.0) [68.6 to 99.4]	34 (83.0) [74.8 to 96.4]	5 (83.3) [40.5 to 126.0]	9 (90.0) [87.8 to 100.8]
Caregivers adherence with CHWs treatment regimen	(N = 55)	(N = 35)	(N = 31)	(N = 14)
Sought care for child’s fever within 24 h of onset of fever	38 (44.2) [33.5 to 54.8]	33 (94.2) [86.0 to 99.7]	20 (64.5) [45.5 to 72.9]	11 (78.5) [64.5 to 85.4
Caregiver gave correct dose of LA to fever child	10 (37.0) [18.1 to 55.9]	24 (68.5) [59.1 to 80.6]	8 (25.8) [17.3 to 36.5]	7 (50.0) [37.2 to 63.2]
Caregiver gave first dose of LA the same day to child	7 (10.4) [2.9 to 17.9]	35 (100.0) [96.6 to 108.7]	3 (9.6) [3.2 to 17.5]	12 (85.7) [62.6 to 95.1]
Children referred by CHWs (VCs)	(N = 42)	(N = 35)	(N = 36)	(N = 40)
Children referred to Health Facilities from village clinics due to danger signs including severe malaria	11 (26.19) [12.3 to 40.1]	5 ( 5.2) [0.8 to 12.3]	4 (11.1) [0.3 to 21.9]	12 (30.0) [15.2 to 46.8]

## Discussion

The findings from this study show that CHWs’ capabilities to perform RDTs, administer pre-referral rectal artesunate and refer patients were relatively high; 97 % of RDTs tests were correctly performed by CHWs and 95 % of sharps and biohazard waste were correctly disposed of. The integration of RDTs into the iCCM programme was also associated with increased adherence to the malaria policy. Overall, the results show that 95 % of CHWs adhered to RDTs results when giving treatment for malaria during the observation study. A review of sick child registers to assess CHWs’ adherence to non-treatment of negative RDT cases showed that 11 % of RDT-negative patients were treated with anti-malarial. This relatively high adherence to RDT results corresponds to results found elsewhere [11] and compares favourably to the behaviour of other health worker cadres in response to negative malaria test results [12, 13]. This illustrates that, when appropriately trained, CHWs can deliver services at the community level at a quality comparable to that delivered by facility-level health workers [14, 15].

The integration of RDTs and pre-referral rectal artesunate also had a positive impact on the utilization of iCCM services. One year after introducing RDTs and pre-referral rectal artesunate, the proportion of caregivers who sought care from CHWs, who promptly (i.e., within 24 h) sought care for a child’s fever, and who received an appropriate anti-malarial following fever/confirmed malaria significantly increased in the intervention villages from 36 to 86.7, 44.2–94.2, and 60–97.2 %, respectively. Care-seeking for cough or pneumonia increased significantly from 29 to 38 % in the intervention villages compared to the control villages, suggesting that the availability of RDTs and rectal artesunate attracted caregivers to seek other iCCM services. The results also show a corresponding sharp decrease in the proportion of caregivers who sought care from other informal sources of care, such as private vendors, shops and traditional healers from 12.9 to 1.9 %. Continued use of shops or vendors for anti-malarials is directly related to incorrect dosing, low drug-adherence levels and possible development of drug resistance [16]. This increase in the utilization of services following integration is in line with global child health strategies and can positively contribute towards the reduction in child morbidity and mortality [17].

This study has also shown high levels of acceptance by both caregivers and CHWs of the integration of RDT and pre-referral rectal artesunate into iCCM services. However, the success of this intervention is very dependent on the availability of CHWs in their catchment areas to provide care when needed. In this study caregivers complained of services being scheduled for certain days and being provided only during the day, mainly because CHWs lived outside their catchment areas or were busy with other engagements. These concerns have previously been documented as obstacles to the scaling up of iCCM services in Malawi [8]. Since CHWs in Malawi are government employees, there is a need to ensure that they live within their catchment areas, thereby enabling them to provide care beyond working hours. Moreover, as the scope of the CHW role is expanding, both in terms of iCCM and other health services, it may be necessary to consider locating two or more CHWs in each hard-to-reach area. This would allow CHWs to alternate between the provision of iCCM or other equally important community-based health services. Furthermore, it is recommended that the Ministry of Health should consider recruiting HSAs within the same hard-to-reach areas or the same districts where they shall provide services. This would deal away with the problem of HSAs residing outside their allocated hard-to-reach areas.

The successful roll-out of an effective rectal artesunate programme also depends on the presence of a functional referral system. In the study area, CHWs referred very sick children from the community to the nearest health facility where they could access more advanced treatment and care. However, caretakers complained that the lack of referral slips meant that referred children were not attended to as soon as they arrived at the health facility and had to queue for services like other, non-referred children. Signed referral slips are key motivators and predictors of referral compliance [18, 19]. As pre-referral rectal artesunate is rolled out, it is important that factors that affect the referral process, such as distance, cost and the lack of referral slips, be addressed to allow timely access to life-saving care and treatment. The issue of referral could be addressed through community sensitization, targeting both caregivers and health workers and focusing on the importance of completing an initial referral slip and subsequent referral feedback slip, the latter being handed back to CHWs who referred the child to the health facility. To encourage referral, a system to remind caretakers to complete referral would be necessary; such reminders have been shown to increase referral compliance [20].

There are some limitations of this study. First, for the quality of care assessment, the fact that the CHWs were being observed during the assessment and were aware of when the assessment would be conducted (they were all advised to conduct iCCM activities during the week of assessment) may have induced them to provide better care than normal. Also, the CHWs providing the services had all received the iCCM refresher training 6 months before assessment, which could have been the reason for the good quality of care results seen.

## Conclusion

These findings indicate that the integration of RDT and pre-referral rectal artesunate at community level is both feasible and acceptable. The strategy has the potential to increase and improve utilization of child health services in hard-to-reach areas and reduce child morbidity and mortality at community level. However, this depends on the availability of these services 24 h a day and 7 days a week in hard-to-reach areas. National iCCM programmes should ensure that CHWs reside in their catchment areas and that adequate resources, including time, are dedicated to supporting the iCCM programme.

## Abbreviations

ACT: artemisinin-based combination therapy
CHWs: community health workers
COM: College of Medicine
COMREC: College of Medicine Research and Ethics Committee
DHMT: district health management team
DHO: district health office(r)
FGDs: focus group discussions
HIV: human immunodeficiency virus
HMIS: health management information system
HSAs: Health Surveillance Assistants
iCCM: integrated Community Case Management
IMCI: integrated management of childhood illnesses
KIIs: key informant interviews
MAC CDAC: Malaria Alert Centre-Communicable Disease Action Centre
RDTs: rapid diagnostic tests
NMCP: National Malaria Control Programme
ODK: open data kit
PPS: probability proportional to size
RA: rectal artesunate
WHO: World Health Organization
